# Immune Checkpoint Inhibitor-related Pancreatitis: A Case Series, Review of the Literature and an Expert Opinion

**DOI:** 10.1097/CJI.0000000000000472

**Published:** 2023-05-23

**Authors:** Sjoerd Kramer, Koen van Hee, Hans Blokzijl, Frans van der Heide, Marijn C. Visschedijk

**Affiliations:** * Department of Gastroenterology and Hepatology, University Medical Center Groningen, Groningen, The Netherlands; † Department of Gastroenterology and Hepatology, Jeroen Bosch Hospital, GZ ‘s-Hertogenbosch, The Netherlands

**Keywords:** immune checkpoint inhibitors, autoimmune pancreatitis, chronic pancreatitis, steroid dependent, steroid failure

## Abstract

Immune checkpoint inhibitors (ICIs) have revolutionized the treatment of various malignancies, but are associated with serious adverse events like pancreatitis. Current guidelines are limited to the first step in treating acute ICI-related pancreatitis with steroids but lack treatment advices for steroid dependent pancreatitis. We describe a case series of 3 patients who developed ICI-related pancreatitis with chronic features such as exocrine insufficiency and pancreatic atrophy at imaging. Our first case developed after treatment with pembrolizumab. The pancreatitis responded well after discontinuation of immunotherapy but imaging showed pancreatic atrophy and exocrine pancreatic insufficiency persisted. Cases 2 and 3 developed after treatment with nivolumab. In both, pancreatitis responded well to steroids. However during steroid tapering, pancreatitis recurred and the latter developed exocrine pancreatic insufficiency and pancreatic atrophy at imaging. Our cases demonstrate resemblances with autoimmune pancreatitis based on clinical and imaging findings. In line, both diseases are T-cell mediated and for autoimmune pancreatitis azathioprine is considered as maintenance therapy. Guidelines of other T-cell mediated diseases like ICI-related hepatitis suggest tacrolimus. After adding tacrolimus in case 2 and azathioprine in case 3, steroids could be completely tapered and no new episodes of pancreatitis have occurred. These findings support the idea that the treatment modalities for other T-cell mediated diseases are worthwhile options for steroid dependent ICI-related pancreatitis.

Immune checkpoint inhibitors (ICIs) are a large breakthrough in the field of oncology and are widely used for the treatment of several malignancies. These checkpoint inhibitors are directly involved in the enhancement of the anti-cancer response and target the molecules cytotoxic T-cell lymphocyte-4 (CTLA-4), programmed death-1 (PD-1) and programmed death-ligand 1. However, the ICIs are associated with different types of immune-related adverse events (irAEs). We present three cases of ICI-related pancreatitis with a different presentation and treatment strategy. Furthermore, we will give an overview of the literature and different therapeutic approaches for steroid failure.

## CASE PRESENTATIONS

### Case 1

**FIGURE 1:**
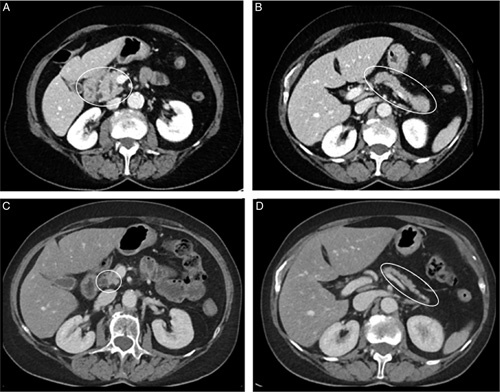
Follow-up CT of the pancreas. During presentation with abdominal pain and high lipase (A and B) CT showed an acute pancreatitis with and edematous head (A) and tail (B) of the pancreas. Five months later the pancreas appears to be atrophic (C; pancreatic head, D; pancreatic tail) without signs of inflammation.

A 74-year-old female patient was treated with pembrolizumab (anti-PD-1) every 6 weeks for 2 years for metastasized lung cancer. There were no signs of disease progression and therapy was stopped. 8 weeks later, she presented with epigastric pain and nausea. Laboratory results showed an elevated serum lipase of 1982 U/l (<393 U/L). CT-imaging of the pancreas showed swelling of the pancreatic head and tail including a capsule-like rim around the tail (Fig. 1). After exclusion of other causes of pancreatitis, i.e. alcohol, gall stones, medication, hypertriglyceridemia, hypercalciemia and IgG4-disease, an ICI-related pancreatitis was diagnosed. No treatment was started and lipase normalized to 65 U/L spontaneously after 4 weeks. The pancreatitis was mildly predicted with a Ranson score of 0 and no admission was required. Since pain persisted after every meal, combined with weight loss and loose and frequent stools, the patient was referred to the gastroenterology outpatient clinic. Fecal elastase was low (5 µg/g (>200 µg/g)) and calprotectin was normal. Additional sigmoidoscopy did not show signs of colitis. Exocrine pancreatic insufficiency was diagnosed and pancreatic enzymes were prescribed, after which complaints disappeared. CT-imaging was repeated which showed atrophy of the pancreas (Fig. 1). This case shows an acute ICI-related pancreatitis with subsequent progression to pancreatic atrophy and exocrine insufficiency. There were no signs of pancreatic endocrine dysfunction.

### Case 2

**FIGURE 2:**
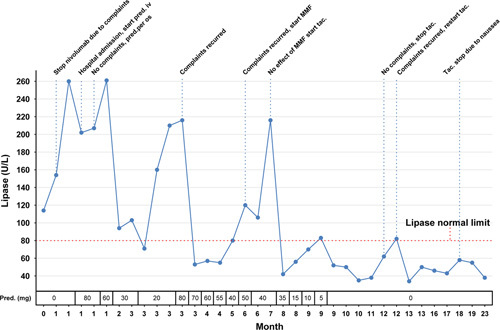
An overview of serum lipase in time and clinical decisive moments. The course of complaints, serum lipase and therapeutic interventions is depicted in this figure. The patients complaints and serum lipase responded well on prednisolone but recurred after tapering despite adding mycophenolate mofetil. However, after adding tacrolimus, prednisolone could be completely stopped. After two attempts, tacrolimus could also be completely stopped and thus far no new episodes of pancreatitis has occurred. Nivolumab is permanently stopped. MMF, mycophenolate mofetil; pred, prednsilone; tac, tacrolimus.

A 41-year-old female patient treated with nivolumab (anti-PD-1) every 4 weeks and radiotherapy after surgical resection of a cerebral metastasized melanoma, presented with epigastric pain and nausea, 8 months after starting nivolumab. The laboratory results showed slightly elevated serum amylase of 147 U/L (<107 U/L) and lipase of 260 U/L (15-80 U/L). CT-imaging of the abdomen showed edema of the caput of the pancreas. An acute ICI-related pancreatitis with Ranson score 0 was diagnosed, after exclusion of alternative causes of pancreatitis, including IgG4 disease. The patient was admitted, nivolumab was interrupted and treatment with 1 mg/kg prednisolone iv was started. Initially, her complaints resolved within 5 days, serum lipase normalized and after 7 days she could be discharged from the hospital. Two months afterwards, CT of the pancreas showed no abnormalities. However, during two attempts of tapering prednisolone (Fig. 2), recurrence of symptoms and an increase of serum lipase occurred. In the literature, there are only case reports describing discontinuation of immunotherapy and high dose steroids for treating ICI-related pancreatitis (see Table, Supplemental Digital Content 1, http://links.lww.com/JIT/A738). In comparison with autoimmune type 1 pancreatitis and ICI-related hepatitis, treatment with respectively mycophenolate mofetil^1^ and tacrolimus^2^ was considered. However, despite adding mycophenolate mofetil 1000 mg twice daily, the pain worsened and the serum lipase further increased (to 216 U/L) within 4 weeks. Consequently mycophenolate mofetil was replaced by tacrolimus with a therapeutic drug level between 5 and 7 µg/L. Within 2 months the pain improved and the serum lipase normalized in 5 months. Prednisolone could be completely stopped. One month later, tacrolimus was tapered within a few weeks. Unfortunately, after a few weeks the complaints relapsed and the serum lipase levels increased, therefore treatment with tacrolimus was reintroduced. Second attempt to stop tacrolimus was successful. 1.5 years after the diagnoses of ICI-related pancreatitis she did not have any complaints and lipase was normal. Figure 2 shows an overview of the serum lipase in time and clinical decisive moments.

### Case 3

**FIGURE 3:**
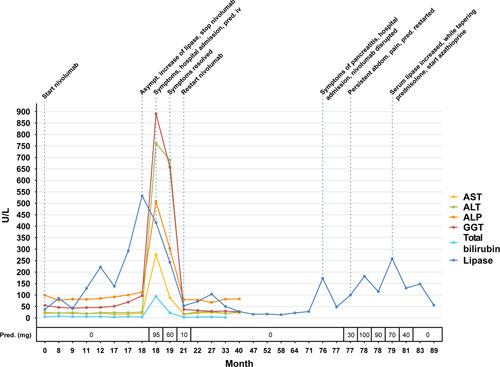
An overview of serum lipase and liver enzymes in time and clinical decisive moments. The course complaints, serum lipase and liver enzymes is depicted in this figure. The first episode of ICI-related pancreatitis occurred with concomitant ICI-related hepatitis and was successfully treated with prednisolone. Afterwards nivolumab was re-introduced without problems. Four years later the patient developed a new episode of ICI-related pancreatitis with chronic features. The patient responded well an prednisolone but the pancreatitis recurred despite two attempts of tapering. After adding azathioprine as maintenance therapy, prednisolone could be completely tapered. Nivolumab is permanently stopped. Pred, prednisolone.

A 56-year-old male patient with progressive grade IV glioblastoma after craniotomy and chemoradiotherapy was included in a phase III trial (CA209143) for nivolumab every 2 weeks. After 38 cycles, with ongoing response, serum lipase gradually increased to 532 U/L (15–80 U/L). Despite having no symptoms, nivolumab was discontinued. However 4 weeks after the last dose the patient developed nausea, loss of appetite, 10 kg weight loss and elevated liver enzymes (AST 277 U/L (<35 U/L), ALT 761 U/L (<45 U/L), ALP 508 U/L (<115 U/L), GGT 891 U/L (<55 U/L), total bilirubin 94 µmol/L (<17 µmol/L)) and a serum lipase of 416 U/L (15-80 U/L). MRCP showed an edematous caput of the pancreas and dilation of the common bile duct (11 mm) with tapering towards the pancreas. After exclusion of other causes of pancreatitis (including IgG4-disease) and hepatitis, a mild ICI-related pancreatitis with Ranson score 2 and ICI-related hepatitis was diagnosed for which treatment with 1 mg/kg prednisolone was started. After 6 days of hospital admission, the patient could be discharged while tapering prednisolone per os. Within 2 months, symptoms resolved and laboratory values normalized. Prednisolone could be completely tapered and nivolumab was reintroduced. 4 years after the restart of nivolumab, the patient developed a mild pancreatitis (Ranson score 1) with nausea, vomiting, a serum lipase of 173 U/L and normal liver enzymes. Nivolumab was discontinued and during admission prednisolone 1 mg/kg was reintroduced. Initially the symptoms and lipase improved and after 3 days the patient could be discharged. However despite three attempts to taper prednisolone, lipase increased, MRI of the pancreas showed atrophy without acute inflammation and fecal elastase was decreased (55 µg/g (>200 µg/g)) indicating exocrine pancreatic insufficiency. There were no signs of endocrine pancreatic insufficiency. Because of the clinical resemblances with autoimmune pancreatitis, azathioprine was added as maintenance therapy. Subsequently, the lipase levels dropped and prednisolone could be completely reduced within 6 months. So far, our patient still uses azathioprine and nivolumab is permanently stopped. No new episodes of pancreatitis have occurred and there are no signs of disease progression of the glioblastoma. Figure 3 shows an overview serum lipase and liver enzymes in time and the clinical decisive moments.

## DISCUSSION

Currently the incidence of ICI-related pancreatitis is low: 0.9–3% for anti-CTLA-4, 0.5–1.6% for anti-PD-1 and 1.2–2.1% for anti-CTLA-4 and anti-PD-1 combined.^2^ An elevated lipase has been reported in 2.3% of the patients receiving anti-PD1 or anti-PDL-1, though only 14% of them developed clinical or radiologic signs of real pancreatitis.^3^ Like autoimmune pancreatitis, ICI-related pancreatitis is considered a T-cell-mediated process. The checkpoint inhibitors act by blocking inhibitory signals on T cells leading to tumor cell death. These overactive T cells with an increased CD8^+^/CD4^+^ ratio may cause damage to pancreatic cells leading to a decrease in pancreatic endocrine or exocrine function and sometimes to peripancreatic fat infiltration and pancreatic atrophy.^4^ Although ICI-related pancreatitis shows similarities with autoimmune pancreatitis, in case reports, IgG4 is reported within the normal range.^5^ There are case reports in which patients develop histological proven IgG4-related pancreatitis^6^ and cholangitis after nivolumab.^6,7^ However in our three cases IgG4 was normal, confirming the data in the literature on usually normal serum IgG4 levels in ICI-related pancreatitis.

ICI-related pancreatitis may present like other cases of traditional acute pancreatitis, but can also have a more atypical presentation. In the current literature ICI-related pancreatitis is diagnosed on the same diagnostic criteria as other types of acute pancreatitis.^4^ ICI-related pancreatitis is graded from scale I–V, in which grade I represents an amylase or lipase level of ≤1.5 times the upper limit of normal (ULN), grade II >1, 5–2 × ULN, grade III >2–5 × ULN, grade IV >5 × ULN and grade V resulting in death.^4^ In case of acute pancreatitis, ICI-related pancreatitis generally appears on CT-scan with characteristics of an interstitial edematous pancreatitis with pancreatic enlargement, decreased enhancement and surrounding fat stranding. However, imaging features of ICI-related pancreatitis are nonspecific and could mimic autoimmune and chronic pancreatitis. In addition, in the early phase chronic pancreatitis is hardly seen on CT or ultrasound but sometimes fibrosis, diminished enhancement and pancreatic duct enlargement could be seen on MRI/MRCP.^8^ We describe 3 cases of a chronic pancreatitis showing similarities to autoimmune pancreatitis. Two of them developed exocrine pancreatic insufficiency and pancreatic atrophy. Fifteen percent of the patients with ICI-related pancreatitis develop long term adverse events like recurring acute pancreatitis, chronic pancreatitis and pancreatic insufficiency and sometimes pancreatic atrophy at imaging.^8^

For asymptomatic isolated elevation of lipase or amylase with or without radiological features of pancreatitis, i.e. grade I ICI-related pancreatitis, immunotherapy can be continued with close monitoring of symptoms.^9^ The next step in treatment regimen, i.e. symptomatic grade I or grade II ICI-related pancreatitis (moderate), is discontinuation of immunotherapy and prednisolone/methylprednisolone at 0.5–1 mg/kg.^2–4,9^ After successful treatment without clinical or radiological signs of pancreatitis, a rechallenge with immunotherapy could be considered.^9^ For grade III–IV ICI-related pancreatitis, prednisolone/methylprednisolone at 1–2 mg is indicated and immunotherapy should be discontinued permanently.^4,9^ The risk of developing the same side effects after restarting immunotherapy is 26–48%, in which 61–78% experienced the same side effect that previously led to discontinuation of immunotherapy.^10^ In a systematic review and meta analyses, ICI-rechallenge showed the same efficacy compared to initial treatment.^11^ Regarding a rechallenge the following approaches can be considered: a rechallenge with the same immunotherapy after resolution of the side effects, switching from ICI-combination therapy to ICI-monotherapy, a class switch from anti-PD-(L)1 to anti-CTLA-4 or vice versa, and a rechallenge with concurrent immunosuppression.^10,12^ The rechallenge strategy depends on actual status of the tumor, patients preference, the risk and benefits of recurring irAEs and alternative treatment options. Currently the data regarding a rechallenge strategy is limited to small number and includes predominantly retrospective studies, case reports and expert opinion papers. There are no studies regarding the rechallenge strategy in ICI-related pancreatitis. We would argue for rechallenge since in general ICI-related pancreatitis does not appear to be associated with mortality^13^ and exocrine insufficiency can be treated by pancreatic enzymes.

It is challenging to consider the effect of immune suppressive agents on the anti-tumor function of immunotherapy. irAEs have been associated with better tumor outcomes because it may indicate a better immune response,^14^ but treating irAEs may affect the efficiency of ICIs. In vitro, dexamethasone impairs the activation and killing ability of tumor-infiltrating lymphocytes, however after withdrawal this function restored.^15^ In mice, early use of corticosteroids shows signs of tumor regrowth, because of a lasting impaired anti-tumor response with reduction of CD8^+^ memory T cells.^16^ When corticosteroids were administered later, i.e. after tumor regression, this effect was not seen. Some studies suggest that patients receiving corticosteroids when starting ICIs had decreased overall and progression free survival. However, the indication for steroids, i.e. comorbidities and tumor burden, could bias the outcome.^17^ In 16 retrospective studies after performing subgroup analysis on corticosteroids administrated for irAEs, no effect on overall survival was seen.^18^ Considering all together from a review from 2022, corticosteroids do not seems to have a large negative effect on survival.^17^ For azathioprine, there is no literature on how agents like this affects anti-tumor response.^17^ In vitro, low dose tacrolimus (<1 ng/mL) does not influence T-cell function. Higher dose tacrolimus impairs T-cell function and tumor killing comparable with dexamethasone.^19^

In our case series we describe 2 steroid-dependent patients with ICI-related pancreatitis. In the current literature there is no advice on treatment of steroid dependent ICI-related pancreatitis. Pathophysiologically, ICI-related pancreatitis shows an increase in CD3^+^ and CD8^+^ lymphocytes like ICI-related hepatitis^20^ In line, autoimmune pancreatitis type 1 could present with CD8^+^ lymfocytes in the pancreatic parenchyma.^21^ In autoimmune pancreatitis type 1, when there is relapse after tapering of prednisolone, azathioprine and mycophenolate mofetil are considered.^1^ Based on data on ICI-related liver injury, it is worthwhile to add mycophenolate mofetil or tacrolimus as maintenance therapy. Based on these findings and our case series, these are the available options in steroid dependent ICI-related pancreatitis.^2^

## CONCLUSIONS

ICI-related pancreatitis can present as an acute pancreatitis. However, there must be awareness that some cases act as a chronic autoimmune like pancreatitis, with the comparable clinical and radiological features as recurrent abdominal pain, pancreatic insufficiency and pancreatic atrophy. Based on our case series, overview of the literature and the guidelines for comparable T-cell mediated diseases as autoimmune type 1 pancreatitis and ICI-related liver injury we demonstrate that azathioprine and tacrolimus are good treatment options for steroid dependent ICI-related pancreatitis.

## CONFLICTS OF INTEREST/FINANCIAL DISCLOSURES

All authors have declared that there are no financial conflicts of interest with regard to this work.

## Supplementary Material
