# Supplementary material for: Immune Checkpoint Inhibitor-related Pancreatitis: A Case Series, Review of the Literature and an Expert Opinion
Source: J Immunother. 2023 May 23;46(7):271–5. doi: 10.1097/CJI.0000000000000472 (PMC10405787; doi:10.1097/CJI.0000000000000472)
Supplement: Supplementary file 1 [file cji-46-271-s001.docx]

**Supplemental Digital Content 1**Table with an overview of cases described in current literature.
ANA, Antinuclear Antibodies; CBD, common bile duct; ERCP, endoscopic retrograde cholangiopancreatograph; EUS, endoscopic ultrasound; FDG, Fluorodeoxyglucose.; Ig, Immunoglobulin; FNA, fine needle aspiration; FNB, fine needle biopsy; SCC, squamous cell carcinoma.

| **Tumor type/patients** | **Immunotherapy regimen** | **Time of onset** | **Symptoms** | **Laboratory findings** | **Imaging** | **ICI-related pancreatitis therapy** | **Outcome** |
| --- | --- | --- | --- | --- | --- | --- | --- |
| Lung adenocarcinoma. Female, 66 y. Ikeuchi et al; 2015.^22^ | Nivolumab 3 mg/kg every 2 weeks | 18 days     28 days | Anorexia, vomiting and back pain | Initially grade 3 elevation of lipase and amylase  Grade 4 elevation of lipase and amylase accompanied by grade 4 increase in AST, ALT and creatinine | Initial CT and MRI showed no pancreatic abnormalities  On day 41, follow-up MRCP showed slight pancreatic swelling | Initially intravenous fluid therapy    Initially prednisolone 1 mg/kg. Eventually, 4 mg/kg prednisolone was required and could gradually be tapered | On day 51 she had no symptoms and lipase and amylase normalized |
| SCC (stage IIA). Male, 64 y. Jiang et al; 2018.^23^ | Nivolumab 140 mg | 1 day | Unbearable abdominal pain | Amylase 253 U/L | Obvious exsudative lesions around the pancreas (CT) | Comprehensive therapy (not specified) | Death 5 days after submission (1 day after sudden unconsciousness) |
| Melanoma (stage III). Male, 63 y. Dehghani et al; 2018.^24^ | Nivolumab | 15 months       18 months | Asymptomatic       12 kg weight loss, diarrhea | Initially normal lipase (63 U/L), normal IgG4     Hyperglykemia (11 mmol/L), lipase > 3x normal, liver enzymes > 10 x normal, fecal elastase <15 µg/g | Peripancreatic fatty infiltration (CT), intense focal uptake (PET) and restricted diffusion of the pancreatic tail (MRI)  Decreased pancreatic volume (50%) | Continuation of therapy      Nivolumab was discontinued earlier (satisfactory response), diabetic treatment, pancreatic enzyme suppletion | Complete response of primary melanoma, with new metastases (left arm and brain). Insulin dependent |
| Melanoma. Male, 58 y. Kohlmann et al; 2019.^25^ | Nivolumab | 4 months | Belt shaped epigastric pain | 5 days before the first symptoms: lipase 82.2 U/L, amylase normal   Onset of symptoms: lipase 394.2 U/L, amylase 318 U/L | Equivocal anechoic inflammation pancreas (ultrasound) and edematous swelling within the pancreatic tail (CT) | A total of 5 courses methylprednisolone and eventually slow tapering of prednisolone (6.5 months) | No signs of exocrine or endocrine pancreatic insufficiency |
| Renal cell carcinoma (stage IV). Female, 70 y. Tanaka et al; 2019.^26^ | Nivolumab | 6 months | Not described | Amylase 547 U/l; lipase 1830 U/L | Mimicking autoimmune pancreatitis. Diffuse enlargement of the whole pancreas (T2 MRI and EUS). restricted diffusion in the pancreatic head and body (MRI). Skipped narrowed laesions of the main PD in the pancreatic head and body (ERCP) | Discontinuation of nivolumab for 2 months | Pancreatic enzymes normalized and enlargement of the pancreas on CT disappeared |
| Melanoma. Male, 79 y. Song et al; 2021^27^ | Nivolumab | 12 months | Jaundice, no abdominal pain | Normal lipase, markedly elevated liver function tests | Hypermetabolic activity at the pancreatic head (PET), signs of chronic pancreatitis (EUS) with lymphocyte infiltration surrounding the ducts (FNB), stricture of the lower CBD (MRCP) | Prednisolone tapered | Improvement in stricture of the CBD and normalization of liver enzymes |
| Melanoma. Woman, 37y. Janssens et al; 2021.^28^ | Nivolumab | Unknown | Epigastric pain for 4 months. No other complaints | Lipase 439 U/L; amylase 136 U/L; normal IgG4 | Heterogeneous parenchymal enlargement of the pancreatic body (CT) and PET-active. Bulky pancreatic body and tail (EUS) with neutrophil infiltrate and fibrosis without IgG4-positive plasma cells (FNB) | Prednisolone 40 mg for 4 weeks, afterwards slowly tapered in three months | Symptoms revolved after 2 weeks, lipase normalized after 6 weeks |
| Melanoma (stage IIIC). Male, 58 y. Kohlmann et al; 2019.^25^ | 4 cycles of Ipilimumab/nivolumab followed by monotherapy nivolumab | 106 days | Epigastric pain | Lipase 394.2 U/L (5 days earlier 82.2 U/L); amylase 318 U/L | Equivocal anechoic inflammatory areas (sonography) and edematous swelling at the pancreatic tail (CT) | Discontinuation of immunotherapy and initiating steroids, slowly tapered in 6.5 months | Complete response of melanoma. No signs of exocrine or endocrine pancreatic insufficiency |
| Melanoma. Male, 60 y. Goyal et al; 2020.^29^ | Ipilimumab/nivolumab followed by monotherapy nivolumab | 4 cycles + 3 cycles of nivolumab | Mild abdominal pain | Lipase 517 U/L. Normal ANA and IgG | Diffuse FDG-avidity of the pancreas with peri-pancreatic fat stranding (PET). 3 weeks later: diffuse mild enlargement of the pancreas (CT) and dilated extrahepatic bile duct of 11 mm with an intrahepatic stricture (MRCP). EUS-FNA of the pancreatic head showed chronic pancreatitis | Intravenous fluid therapy and prednisolone (tapered in 14 days) | Three weeks after completely tapering prednisolone, abdominal pain worsened and cholestatic obstructive lab results. After a high dose prednisolone with a prolonged tapering period the lab results normalized and PET showed resolution of pancreatitis |
| Melanoma (stage III). Male, 76 y. Yamamota et al; 2021.^30^ | Nivolumab 80 mg and ipilimumab 150 mg | 3 weeks after the second cycle | 2 week fatigue, anorexia and diarrhea. No abdominal pain | Initially normal amylase and lipase. On the 4^th^ day of admission: amylase 683 U/L; lipase 1520 U/L; CRP 31 mg/dL; normal IgG4 | Enlarged pancreatic parenchyma (CT) and peripancreatic fat stranding (MRCP) | 0.5 mg/kg prednisolone | Symptoms improved |
| Lung adenocarcinoma. Male, 46 y. Das et al; 2019.^31^ | Pembrolizumab | 3 cycles | Epigastric pain | Amylase and lipase were elevated | Focal FDG uptake in the pancreatic tail (PET) corresponding to focal region of pancreatic tail enlargement with mild surrounding mesenteric stranding (CT) | Discontinuation of pembrolizumab and initiating steroids | Repeat PET-CT at 3 months showed resolution of FDG uptake and pancreatic tail enlargement. Pain resolved and lipase normalized |
| Squamous cell lung carcinoma. Male 70 y. Kakuwa et al. 2020.^32^ | Pembrolizumab, 200 mg every 3 weeks | 14 months | Asymptomatic | Grade 2 elevation of lipase and amylase | A PET-CT showed a round-shaped lesion at the pancreatic head and CT showed slightly swollen pancreas and mild pancreatic duct dilatation | Prednisolone 1 mg/kg | 15 days after the initiation of prednisolone, the pancreas showed no abnormalities on PET-CT and CT |
| Metastatic colon cancer. Male, 43 y. Delgado-Lazo et al; 2022.^33^ | Pembrolizumab | 7 months | Shortness of breath and epigastric pain | Elevated troponine at 0.029 ng/mL and Lipase of 588 U/L | Left bundle branch block on ECG and a decreased ejection fraction of 15-20% on echocardiogram. Angiogram showed normal coronary arteries. CT-abdomen showed peripancreatic stranding | A diagnose of ICI-myocarditis and pancreatitis was made and methylprednisolone 125 mg iv was started | The signs and symptoms of myocarditis and pancreatitis improved and the steroids could be tapered. Pembrolizumab was discontinued permanently |
| Melanoma. Male 57 y. Alabed et al; 2015.^34^ | Ipilimumab | 3 cycles | Asymptomatic | Lipase 107 U/L and amylase 107 U/L | Diffuse FDG uptake in the body of the pancreas and fat stranding peripancreatic (PET-CT) | Not described | Not described |
